# Homophily in social and demographic traits predict association patterns in female western and mountain gorillas

**DOI:** 10.1098/rspb.2024.1956

**Published:** 2024-11-27

**Authors:** Christopher Young, Martha M. Robbins

**Affiliations:** ^1^ Department of Psychology, Nottingham Trent University, 50 Shakespeare Street, Nottingham NG1 4FQ, UK; ^2^ Max Planck Institute for Evolutionary Anthropology, Deutscher Platz 6, Leipzig 04103, Germany

**Keywords:** association patterns, cross-species, female dispersal, social network analysis, social relationship

## Abstract

Affiliative relationships are a hallmark of social relationships in gregarious mammals, but what drives variation of association patterns when kin are absent remains unknown. Gorillas, where females may disperse multiple times in their lives, provide an interesting counterpoint to female philopatric species to examine the factors influencing variation in association patterns. We examined demographic and social factors that may predict association patterns of female western (*Gorilla gorilla gorilla*; Loango, Gabon) and mountain gorillas (*Gorilla beringei beringei*; Bwindi, Uganda). We looked at dyadic and individual strength scores of social proximity (37 group-years). For individuals, high dominance rank increased association scores while newly emigrated females had lower scores than resident females. For dyads, higher mean dominance rank and both partners having a dependent infant increased association scores, whereas a partner being an immigrant decreased scores. Furthermore, time-matched analysis of birth and immigration events confirmed the temporal nature of these associations. Overall, female gorilla association patterns show flexibility in strength based on real-time contingencies, namely social and demographic traits. Association patterns in species with female secondary dispersal may be governed by homophily, like that of modern humans. Understanding female gorilla social structure can enhance our knowledge of the evolutionary origins of sociality.

## 1. Introduction

The link between affiliative relationship quality and positive fitness outcomes is well established [1–3]. However, this research has mainly focused on species where females are philopatric, remaining in their natal groups throughout their lives and thus reaping kin-based benefits (maternal or paternal kin, e.g. in primates and other social mammals [4–11]). Owing to the duration of group tenure with kin, the opportunity to invest in long-term affiliative relationships leads to the formation of strong, stable relationships over time [12]. As the formation of strong relationships has been shown to enhance fitness and survival for the individual and their offspring ([2,13,14] but see [15]), it stands to reason that philopatric females would form these relationships with close kin, gaining indirect fitness benefits [16,17].

Nonetheless, not all gregarious species show female philopatry [18,19]. In species where females disperse from their natal groups at maturity, kinship and the indirect fitness benefits of kin selection are likely to play only a minor role in shaping social dynamics. However, differentiated social associations (a range of stronger and weaker associations within a social group) among females are still observed in species with female dispersal. For example, in bonobo (*Pan paniscus*) and chimpanzees (*Pan troglodytes*), females emigrate into new groups at maturity and can form long-term relationships similar to those of philopatric species, but not necessarily with kin [20–23]. As these females only disperse once in their lifetimes, they can gain similar benefits of investing in long-term relationships to species with female philopatry, but they are not governed by kin selection. However, in a few species, females may disperse multiple times in their lives (secondary dispersal), including in social equids, tropical bats and some primates [18,24–28]. Similarly, early hominid and hunter gatherer females are considered to have shown some level of female-biased secondary dispersal [29–32]. Species in which females exhibit multiple dispersal events can provide insights into the evolution of modern human sociality. Females dispersing multiple times throughout their lifetime can affect group stability, cohesion and tenure of individuals in social groups, as well as the potential to form strong long-term affiliative relationships.

Secondary dispersal therefore creates an evolutionary puzzle in relation to social associations among females, as they must invest in social behaviour with partners who could disperse at any time. Hence, the formation of long-term social associations may not be as beneficial or even achievable owing to the social insecurity driven by the fact that one dyad member could emigrate. In these species, female affiliative relationships tend to be weaker than in species with only primary female dispersal [33,34]. However, there is still differentiation in affiliative relationship strength and understanding the factors that drive the formation of stronger relationships in these species with secondary dispersal is important to understand how affiliative relationships have evolved. In such species, it may be that affiliative relationships are underpinned by immediate needs and differing social strategies [35,36]. This could vary depending on the needs of the individuals, availability of desirable or compatible partners and/or environmental or demographic factors. Thus, affiliative relationships might be formed based on short-term contingencies [35,36] driven by similarity between potential partners. Alternatively, social relationships may be governed by three dimensions: (i) value (the direct benefits gained), (ii) security (the consistency of interaction over time) and (iii) compatibility (tolerance between individuals) over time [37] leading to long-term affiliative relationships as seen in female philopatric species [12].

The similarity hypothesis proposes that females will form relationships with group members expressing similar traits (homophily), such as dominance rank, age or residency length [38–41], because they will be more mutually attracted and share a suite of appealing attributes. For example, in humans, similar characteristics and traits (e.g. age, education, social status and personality traits) have been shown to predict affiliation [41]. This homophily could also relate to needs for infant protection from harassment or predation [42,43], so associating with a group mate experiencing similar risk could be advantageous and lead to increased association in times of need (also see transitory states [36]). In line with the similarity hypotheses, individuals who have similar resource-holding potential, i.e. close dominance rank positions, may associate more frequently owing to similar access to contested resources such as feeding sites, sleeping sites, mating partners or inter-sexual social partners [44,45]. For example, in the more egalitarian crested macaques (*Macaca nigra*), females closer in rank are likely to form social relationships [46]. Therefore, individuals with comparable traits may be more likely to form affiliative relationships when kin are not present.

Individual dispersal decisions may also impact affiliative relationships in species with multiple dispersal events in their lifetimes. Social behaviour can also be linked to the likelihood of an individual emigrating from their current social group, if dispersal decisions are based partially on affiliative relationships. For example, male vervet monkeys (*Chlorocebus pygerythrus*), who show secondary dispersal, are more likely to depart their social group if they are low-ranking or have lower association scores [47]. Consequently, if a group member is likely to disperse from their social group then this could lead to a deterioration of their affiliative relationships with other group members.

Here, we investigate possible factors that predict individual and dyadic association patterns in three mountain gorilla groups (*Gorilla beringei beringei*) and one western gorilla group (*Gorilla gorilla gorilla*) across multiple years. Females of the two species of gorilla show multiple dispersal events throughout their lives (once every 4.5 years on average, but some female dyads may co-reside in a group for >10 years) and have been shown to exhibit differentiated dyadic association patterns [34,48–50]. However, what variables influence the formation and maintenance of these differentiated dyadic associations remains to be examined. The aim of this study is to investigate what is driving the propensity of female gorillas to form stronger social associations with some unrelated group members but not others.

The two gorilla species occupy a vast range of ecological conditions and vary greatly in dietary patterns, but they show many similarities in their social organization [51]. Females may disperse from their natal group at maturity and can transfer to new groups multiple times throughout their lives, so residing with close kin is unlikely ([49,50] but see [52]). Females of both gorilla species have recently been shown to form differentiated spatial associations that can be stable for an average of 2 years [34,48]. These associations were not be driven by females concurrently associating with the alpha male as a means to reduce predation risk and infanticide [49,50,53,54]. However, what drives these associations and the possible factors that would lead certain females to have stronger associations at certain times remain to be investigated.

To understand factors driving female–female social associations in gorillas we examined both individual and dyadic levels of association. First, at the individual level, we aimed to understand if there were general traits that lead to a greater propensity to associate and, second, if there were additional traits that predicted associations between certain dyads. Female association scores were measured from time spent in close proximity (<5 m) on an annual basis. We did not use grooming because it almost never occurs among adult western gorillas [34].

For individuals, we predicted that a female would show higher association levels if she had a dependent infant born that year, as a strategy to minimize risk of infanticide, harassment or predation. Alternatively, she may be drawn to associate more with other mothers to allow her infant to socialize (I1). Also, if the female was a recent immigrant to the group, we predicted she would show lower association scores (I2), as a new immigrant may be apprehensive to spend time close to others owing to risk of receiving aggression [55]. Additionally, if she were to leave the group the following year we predicted she would show lower association scores as she may invest less in group activity (I3), as seen in other primates [47]. Higher-ranked individuals may show greater association owing to prioritized access to high-quality resources (preferable feeding or resting sites) and so are more likely to be in mutual proximity, enhancing their association score (I4). However, we may see the opposite in mountain gorillas, where high-ranking females have been observed to feed more frequently alone (I5 [56]). Group instability caused by alpha male turnover may also play a role in disrupting association patterns. Owing to the role of the alpha male as a social hub in gorilla society [49,50,53,54], females may reduce time spent in association with each other to forge and strengthen relationships with a new alpha male (I6).

Second, we aimed to understand female dyadic association and its predictors. Here, we predicted that the similarity hypothesis would play an important role, and females were more likely to associate if they (i) both had a dependent infant (D1) and (ii) were both higher-ranked (D2: average dyadic rank score). We predicted that association scores were likely to be lower if one dyad member was a recent immigrant (D3). As above, we predicted that social instability (alpha male turnover) would lead to a decrease in dyadic female association (D4). Previous research on the same populations showed similar patterns of associations in both mountain and western gorillas [34], and thus we did not predict specific differences in the predictors of associations but explored possible differences between the species for the first time.

Finally, we aimed to examine the temporal change in meaningful predictors of association at the dyadic level from the above static analysis. For any meaningful predictors (infant births, immigrant arrival, alpha male turnover) we took a time-matched dynamic approach to investigate how association scores changed over time in relation to the time of the event in comparison to other group dyads during a particular year, and two years before and after this. We predicted that, for the meaningful events, those dyads who experience that event (e.g. a birth, alpha male turnover or immigrant arrival) would show a greater change in association scores compared to their time-matched conspecifics.

## 2. Methods

### (a) Study site and subjects

Behavioural observations were conducted on three groups of habituated mountain gorillas in Bwindi Impenetrable National Park, Uganda. The Kyagurilo (KYA) group was observed from 2001 to 2019, the Bitukura (BIT) group was observed from 2015 to 2019 and the Oruzogo (ORU) group was observed from 2015 to 2019. One group of habituated western gorillas in Loango National Park, Gabon (Atananga Group; ATA) was observed from 2015 to 2022 (for demographic information refer table 1). Both ORU and BIT were multimale throughout the study period, KYA was both one-male and multimale, while ATA was always one-male. All research assistants collecting data were trained and supervised on a routine basis by MMR to ensure uniformity in data collection. Females were considered as adult when aged 10 years or older and no known mother–adult daughter, full or half-sibling pairs were co-residing during the study period.

**Table 1.** Distribution of demographic data and predictor variables among the three mountain (KYA, BIT and ORU) and one western (ATA) gorilla groups.

group	data collection period	number of:						
		individual females (yearly mean and range)	focal observations per female per year (mean ± s.d.)	unique dyads	immigrant females	infants born	alpha male changes	departing females
KYA	2001–2019	12 (6.2; range = 5–7)	104.46 ± 56.90	37	7	15	2	3
BIT	2015–2019	4 (4; range = 3–5)	79.92 ± 38.27	6	0	2	1	0
ORU	2015–2019	5 (4.5; range = 4–5)	91.70 ± 42.71	10	0	5	0	0
ATA	2015–2022	6 (4.5; range 2–7)	157.30 ± 104.22	21	0	5	0	4

### (b) Behavioural data collection

In Bwindi, observations of the gorillas were limited to 4 h per day, as per regulations of the Uganda Wildlife Authority, and typically occurred between 08.00 and 15.00 h. In Loango, observations were conducted all day, typically between 07.00 and 1630 h for an average of 5.7 h per day. Data collection protocols were identical at the two study sites and consisted of focal animal sampling (15−60 min duration) and instantaneous scan sampling (conducted at 10 min intervals) of all adult females (for full methodology refer to [34,57]). Focal durations varied when the focal animal moved out of sight before the end of the full 60 min observation because it is often difficult to follow an individual for an extended time owing to the dense understory, which limits maneuverability and visibility for the observers. The activity of the focal animal (feeding, resting, travelling or other) and proximity of all other group members in view were noted in the scan sampling record. We used a distance of <5 m to the focal animal as a measure of female association [34,52,58].

### (c) Social proximity measure

The female–female social association scores were determined from social proximity. Full details of the method are given by Young & Robbins [34]. Briefly, social matrices (non-directional) were constructed using the 5 m proximity scans from the instantaneous scan sampling using the ‘netTS’ package in R [59]. Social network measures for ‘Strength’ (weighted degree [60]) were calculated using the ‘igraph’ package [61] at the individual and dyadic levels concurrently. We calculated the yearly individual-level strength scores as the total number of times each individual was observed within a 5 m radius of another female group member per year, controlling for the total number of scans collected per female. We calculated dyadic yearly strength scores controlling for the total number of scans of the two dyad members: Strength = *N*_*ab*_ / (*N*_*a* +_ *N*_*b*_), or the number of times both individuals were within 5 m of each other divided by the total number of scans in which each was the focal animal.

### (d) Female and male dominance hierarchy

Each year, we constructed dominance hierarchies separately for males (to determine which adult male was alpha in multimale groups) and females . We followed the methods of Wright *et al*. [62] and Young & Robbins [34], using displacements and avoidances from all occurrence sampling. An Elo-rating method [63] was used to calculate hierarchies for each sex, group and year in R, using the EloRating package [64]. Individuals had a starting value of 1000, *k* was set at 100 and new group members entered the hierarchy at the bottom by setting the ‘innit’ argument to ‘bottom’ (following [62]). We extracted daily scores for each individual, which were then averaged across the year from 1 January to 31 December. This gave an average Elo score for each group member. In general, female rank order rarely changed throughout the study. For males, the highest-ranked individual for each group/year was determined to be the alpha male for further analysis. If a group had only one male that year, then this male was determined the alpha male by default. For females, we standardized the Elo scores (between 1 (highest-ranked) and 0 (lowest-ranked)) per group and year to compare between different group sizes and interaction rates. From these scores, we determined a dyad's average rank score by summing both individuals' standardized ranks and dividing by two to gain the dyadic average rank predictor variable.

### (e) Demographic variables

We calculated several demographic variables as follows. (i) *Immigrant female*: when a new adult female joined a group, she was termed an immigrant female for the first year in the group. As the association scores were yearly measures, this was the first full association period for which data were available for the female, so if they joined midway through the year their first association score would be for the following year period (dichotomous variable detailing whether a female had joined the group in the previous year). (ii) *Last year in group*: for any given year, if a female emigrated in the following year then that final year was considered her last year in the group (dichotomous variable detailing if a female departed from the group in the next year). (iii) *Dependent infant*: a female was deemed to have a dependent infant if she had an infant of less than 1 year old for more than 6 months of a given year (dichotomous variable—did the female have an infant less than 12 months?). (iv) *New alpha male*: was calculated as a measure of whether the alpha male had changed in the previous 12 months (dichotomous variable—did the alpha male change in the previous 12 months?).

### (f) Statistical analysis

To test our predictions, we used a Bayesian multi-level statistical approach. We used the function ‘brm’ from the R package ‘brms’ [65] implemented in R using ‘r-STAN’ [66] to estimate predictors of individual and dyadic female association strength. Linear mixed models (LMMs) were fitted with Hamilton Markov Chains (HMCs) and run in R v. 4.3.1 [67]; additionally, we fitted the ‘student’ family and link = ‘identity’ for our models to provide the best fit. We present summary statistics for posterior means, standard errors (s.e.) and 95% credible intervals (CI) for the main effects. All continuous variables were standardized by subtracting the mean and dividing by two times the standard deviation to facilitate comparisons of the effect sizes across continuous and dichotomous variables [68]. We used weakly informative priors (mean = 0 and s.d. = 1 for all continuous variables), four chains and 4000 iterations [65,69]; this provided a large enough sampling pool to allow for posterior sampling and model convergence. All $\hat{r}$ were <1.1, which indicates that our models converged, while whole posterior predictive checking indicated that no model assumptions were violated [70]. We used the ‘bayes_R2’ function to generate marginal *R*^2^ and conditional *R*^2^ values [71]. To check for multicollinearity, we first ran general linear models without the random effects and examined the variance inflation factors (VIFs) using the R package ‘car’ and the function ‘vif’ [72]. The VIFs were all below 2.58, indicating that multicollinearity was not an issue.

### (g) Predictors of individual-level strength

To examine what predicts individual-level strength scores we adopted the following approach. We used node-level strength score as our response variable and determined individual-level effects per year. We included a range of demographic- and group-level predictor variables in our LLM model (model 1_INDIVIDUAL_: *n* = 211). These factors were (i) dependent infant (I1); (ii) new immigrant (I2); (iii) last year in the group (I3); (iv) dominance rank (I4 and I5); and (v) new alpha male (I6). For the distribution of predictor variables between gorilla groups, refer to table 1. We included group size (total number of weaned adults) and number of females as control variables and group ID as a predictor variable to determine whether there was a group and/or species difference. Individual ID and year were included as random effects.

### (h) Predictors of dyadic-level strength

To examine what predicts dyadic-level strength scores we adopted the following approach. We built a LLM (model 2_DYADIC_: *n* = 480) with dyadic strength score per year as the response variable and included several demographic and group-level predictor variables. These variables were (i) dependent infant (D1: does one or both females have an infant less than 1 year old: both/one/none); (ii) average dominance rank (D2); (iii) new immigrant (D3); and (iv) new alpha male (D4). We included group ID, group size and female number as a predictor and control variables, as above. Dyad members ID1 and ID2, Dyad ID and year were included as random effects.

### (i) Temporal change in association

To further examine dyadic associations at a time-matched temporal level we undertook the following analysis. For predictor variables in model 2_DYADIC_ that were shown to have a meaningful effect on the female association pattern (see below for dependent infant and new immigrant), we re-calculated the strength scores time-matched for dyads where (i) both females had a dependent infant (centred on the date of the birth of the infant by the second female of the dyad) or (ii) one of the females was a new immigrant (centred on the day when a new female joined the group). Rather than constructing association matrices using the date of 31st December to calculate the strength scores as above, for each dyad of interest we used the date of second infant birth or immigrant group entry as the calculation point to construct the association matrices. For each of these events, the association scores for all dyads in the group for that year of the event plus (i) the preceding 2 years and 2 years after (dependent infant) or (ii) 4 years after (immigrant female) were complied. This gave 5 yearly scores per dyad for each event. We then constructed two generalized additive models (GAM) with the same statistical parameters as above and using a time-window approach [73,74] to account for temporal dependencies and a non-linear relationship between the response and predictor variables. Yearly time-matched dyadic association score per event was the outcome variable for both models. For the dependent infant model (model 3_infant_: *n* = 349), the predictor variables were (i) year in relation to event (range −2 years before to +2 years after); (ii) whether or not both members of the dyad had a dependent infant (yes/no); and (iii) the interaction between these two terms; dyad ID was also included as a random intercept. For the immigrant model (model 4_immigrant_: *n* = 237), the predictor variables were (i) year in relation to migration event (0–4 years after); (ii) whether one dyad member was an immigrant (yes/no); and (iii) the interaction between these terms and dyad ID as a random intercept. As female gorilla ranks are generally consistent across years, we did not have enough variation in female average rank scores within dyads to conduct the same analysis for average rank.

## 3. Results

### (a) Predictors of individual-level strength

**Figure 1.**
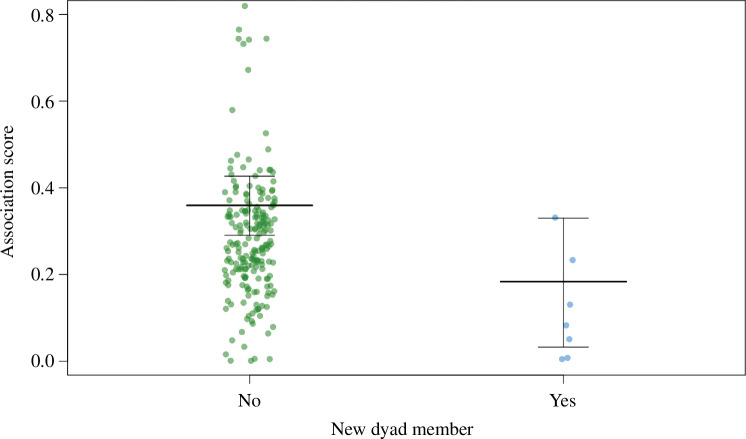
The marginal means and standard error for the relationship between female individual association and immigrant status (raw data points are included as green = no and blue = yes). Results are from the output of model 1_INDIVIDUAL_; for full results refer to table 1.

We found that our demographic- and group-level predictors had an influence on female individual association strength score (table 2). First, there was an independent strong negative influence of whether a female was a new immigrant into the group on female association score, with newly immigrated females (less than 1 year in a group) showing lower association scores (I2: table 2 and figure 1). Second, dominance rank had an independent weak meaningful positive influence, as an individual’s increased dominance rank increased her association score (I4: table 2 and electronic supplementary material, figure S1). Our control variable of group size also had a meaningful positive effect, with larger group size associated with higher association scores (table 2). We found that western gorillas had lower individual association scores than mountain gorillas for one of our mountain gorilla groups (ORU) but not the other two groups (table 2). The variables for dependent infant, tenure length, new alpha male, female last year and female number had no meaningful effect on individual association scores.

**Table 2.** Results of LMM model 1_Individual_ (*n* = 211 individual scores). The estimate, standard error (s.e.) and 95% credible intervals (CI) are provided. *R*^2^_CONDITIONAL_ = 0.722 ( ±0.018 s.e.) and *R*^2^_MARGINAL_ = 0.195 ( ±0.058 s.e.). Factors highlighted in bold are considered to have a meaningful effect on the response variable.

predictors	estimates	s.e.	95% CI
**intercept**	**0.360**	**0.034**	**0.291 to 0.427**
**dominance rank**	**0.033**	**0.015**	**0.003 to 0.065**
dependent infant: yes	0.014	0.014	−0.013 to 0.042
**new immigrant: yes**	**−0.147**	**0.035**	**−0.216 to −0.078**
tenure	−0.016	0.018	−0.052 to 0.019
new alpha male: yes	−0.024	0.029	−0.082 to 0.034
last year in group: yes	−0.019	0.027	−0.072 to 0.034
female number	0.006	0.033	−0.058 to 0.071
**group size**	**0.141**	**0.028**	**0.086 to 0.195**
group: ATA versus BIT	0.045	0.043	−0.039 to 0.134
group: ATA versus KYA	−0.037	0.027	−0.090 to 0.015
**group: ATA versus ORU**	**−0.165**	**0.043**	**−0.249 to −0.079**

### (b) Predictors of dyad-level strength

**Figure 2.**
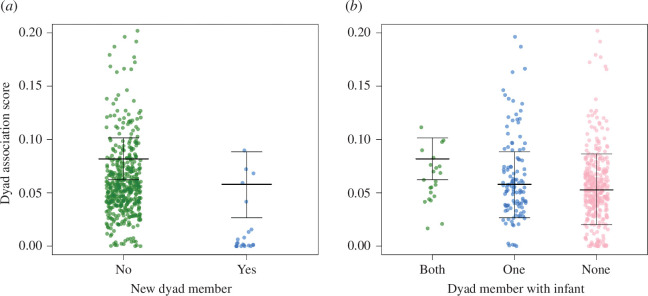
Marginal means and standard error of the relationship between dyadic association score and (*a*) female immigrant status (raw data points are included as green = no and blue = yes), (*b*) whether one dyad member has a dependent infant (raw data points are included as green = both, blue = one and pink = none). Results are from the output of model 2_DYADIC_; for full results refer to table 2.

We found that several of our demographic- and group-level predictors had an influence on female dyadic association score (table 3). First, we found that there was an independent strong negative effect of whether or not one dyad member was a new immigrant on the strength of dyadic association scores; if one dyad member was a new immigrant then the dyadic association was more likely to be lower (D3: table 3 and figure 2*a*). Second, if both dyad members had a dependent infant there was an independent strong positive effect on association score compared to if only one or neither dyad member had a dependent infant (D1: table 3 and figure 2*b*). Third, the average rank of the two dyad members had an independent weak positive effect on female association score: as the average rank of the dyad members increased (i.e. both dyad members were higher-ranking) the dyadic association score was also likely to increase (D3: table 3 and electronic supplementary material, figure S2). Our control variable of female number had a negative effect: as female number decreased, dyadic association score was also likely to decrease (table 3). We saw higher dyadic association scores for the western gorillas compared to the mountain group ORU but no difference between the other two groups. The variables for dependent infant (one dyad member versus none), new alpha male and group size had no meaningful effect on dyadic association scores.

**Table 3.** Results of LMM model 2_DYADIC_ (*n* = 480 dyads). The estimate, standard error (s.e.) and 95% credible intervals (CI) are provided. *R*^2^_CONDITIONAL_ = 0.537 (±0.019 s.e.) and *R*^2^_MARGINAL_ = 0.264 (±0.043 s.e.). Factors highlighted in bold are considered to have a meaningful effect on the response variable.

predictors	estimates	s.e.	95% CI
**intercept**	**0.082**	**0.010**	**0.062 to 0.102**
**dominance rank average**	**0.007**	**0.003**	**0.002 to 0.012**
**new immigrant: yes**	**−0.024**	**0.006**	**−0.036 to −0.013**
new alpha male: yes	0.002	0.006	−0.011 to 0.014
**dependent infant: both versus one**	**−0.017**	**0.006**	**−0.029 to −0.005**
**dependent infant: both versus none**	**−0.016**	**0.006**	**−0.028 to −0.005**
**female number**	**−0.032**	**0.007**	**−0.044 to −0.018**
group size	0.012	0.007	−0.001 to 0.025
group: ATA versus BIT	−0.005	0.012	−0.026 to 0.019
group: ATA versus KYA	−0.002	0.006	−0.015 to 0.010
**group: ATA versus ORU**	**−0.024**	**0.012**	**−0.048 to −0.002**

### (c) Temporal change in dyadic association scores

**Figure 3.**
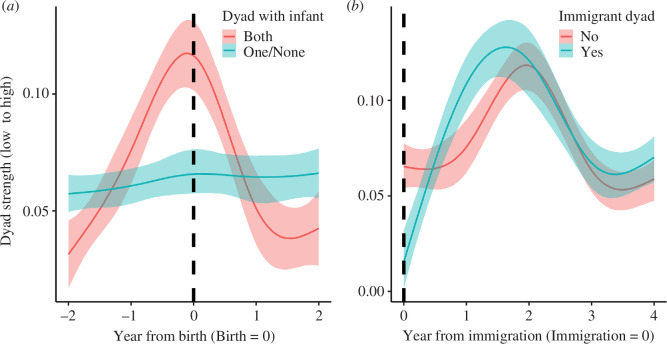
Relationship between association score and (*a*) whether both dyad members had a dependent infant (Both or not (One/None)) and (*b*) female immigrant status (newly immigrated female Yes or No). For the *x*-axes, (*a*) the birth year of the second mother's infant is time zero (dashed line) and the two preceding and proceeding years are shown; (*b*) time zero is the arrival year of a dyad's new immigrant in the group (dashed line) and the proceeding 4 years are shown. For full model results refer to table 4.

When we compared the association score for dyads where both females had a dependent infant (model 3_infant_) to those without, time-matched for date of second birth, we found that there were strong meaningful differences in the interactions both between dyad members having an infant (versus not) and time period, with the peak occurring around the time of the birth event (table 4 and figure 3*a*). For time-matched dyads where neither individual gave birth, we found no meaningful effect. Thus, for dyads where both members had a dependent infant, association scores were likely to rise to a peak at around the time of the infant’s birth and decrease 1 year later, but no such pattern was observed if only one or neither dyad member had a dependent infant. Similarly, when comparing dyads where one member was a newly immigrated female to all other dyads, time-matched for date of immigration event, we found that there was a strong meaningful effect for the interaction between immigrant status and time period, with the lowest scores occurring for newly immigrated dyads (table 4 and figure 3*b*). When considering the year when the female joined the group, these dyads had much lower association scores than all other time-matched dyads and their scores also increased sharply in the subsequent years and were not dissimilar to other time-matched dyads.

**Table 4.** Results of GAM models 3_infant_ and 4_immigrant._ The estimate, standard error (s.e.) and 95% credible intervals (CI) are provided. Model 3_infant_: *R*^2^_CONDITIONAL_ = 0.374 (± 0.040 s.e.) and *R*^2^_MARGINAL_ = 0.170 (±0.030 s.e.); model 4_immigrant_: *R*^2^_CONDITIONAL_ = 0.409 (±0.029 s.e.) and *R*^2^_MARGINAL_ = 0.348 (±0.031 s.e.). Factors highlighted in bold are considered to have a meaningful effect on the response variable. The ‘sds’ parameters indicate the estimated wigglyness of the basis spline.

model	predictors	estimates	s.e.	95% CI
model 3 infant	**intercept**	**0.062**	**0.003**	**0.056 to 0.068**
	**year period × infant: yes**	**0.310**	**0.129**	**0.052 to 0.567**
	year period × infant: none/one	0.030	0.042	−0.077 to 0.154
	sds (year period × infant: yes)	0.600	0.317	0.231 to 2.090
	sds (year period × infant: none/one)	0.045	0.049	0.002 to 0.357
model 4 immigrant	**intercept**	**0.077**	**0.004**	**0.069 to 0.083**
	year period × immigrant: no	0.061	0.089	−0.111 to 0.242
	**year period × immigrant: yes**	**0.802**	**0.112**	**0.573 to 1.022**
	sds (year period × immigrant: no)	0.609	0.317	0.246 to 2.167
	**sds (year period × immigrant: yes**)	**0.518**	**0.256**	**0.225 to 1.649**

## 4. Discussion

We found evidence for predictors of individual and dyadic association patterns in both western and mountain gorillas. At the individual level, we found that higher-ranking individuals were more likely to have higher association scores. We also found that immigration status negatively affected female association scores. If the female had recently immigrated into the group then she showed lower association scores, but association scores did not differ prior to female emigration from a group. At the dyadic level we found support for the similarity hypotheses, with female dyads that both had a dependent infant showing higher association scores than dyads in which only one or neither did. This was further confirmed as a temporal change in association score through the time-matched analysis, as the association scores rose in dyads where both females had dependent infants—starting from initially low average scores and peaking at around the year after birth, before falling again afterwards. This pattern was not observed for time-matched comparable dyads of females with one or no infants. Second, we found weak support for the relationship between dominance rank and association score. The higher the average dominance rank of the dyad (both higher-ranking), the higher their association score. And if one dyad member was a recent immigrant then the dyad was more likely to show lower association scores—thus dissimilar dyads showed lower scores. This was shown to be a temporal negative change in association scores through the time-matched analysis as dyads with one newly immigrated female were lower in the first year the female joined the group compared to subsequent years, and at lower levels than time-matched comparable dyads. As secondary female dispersal is rare in mammals, our study increases our understanding of social behaviour beyond the common well-studied social structures of female philopatry and singular female dispersal by determining the factors diving association between unrelated female gorillas.

Our results show support for the similarity hypothesis. Individuals with similar traits, such as both having a dependent infant [36,38–41], may be drawn together at certain times. In many primate species a new mother can become a social hub and new infants are attractive commodities within social groups [35,75]. For the gorillas, another female with a dependent infant could constitute an attractive association partner because the earliest they will depart the group is when their infant is weaned. In a species with secondary female dispersal such information could be crucial in choosing an association partner guaranteeing a return on their social investment. It is only when both dyad members were new mothers that we saw higher association scores for that dyad, with the scores dropping off in the following years (figures 2 and 3). We propose that these females are associating more frequently for risk aversion purposes because dependent gorilla infants are vulnerable to infanticide and predation [49,50,53,54]. By increasing their association, two new mothers may be able to negate some of these costs and risks to enhance infant survival. While the infants are young and less mobile, mothers may associate for their infants' socialization and a rapid decline in this partnering after 1 year could reflect the infants' increased mobility and preference to socialize close to the silverback [76]. Thus, mothers’ close association is no longer necessary. Strong associations tend to last for 2 years on average and this could be explained by the duration of the life history variables (immigrants integrating after a year [77], immigrants typically giving birth after 1 year or more and having a dependent infant once every 5 years (inter-birth interval 5–5.5 years [78]). So, females only remain similar and thus attractive association partners for short periods during their group tenure, leading to the shorter-term association patterns observed. As highlighted by the time-matched temporal analysis, association scores peaked when both females had given birth and decreased in the proceeding years.

Females of similar rank were shown to be more likely to have higher dyadic association scores and as individual female rank increased the likelihood of having higher association scores also increased. Higher-ranked females may have greater resource-holding potential, being able to gain access to higher quality resources or use their combined higher ranks to defend rare commodities [79]. Female gorilla coalition formation has seldom been observed in the wild [80], but for high ranked dyads their combined presence could be enough to monopolize key clumped food resources (such as fruits), or access to the alpha male as a grooming partner. Indeed average food site residence times were correlated with dominance rank for female mountain gorillas in the Virungas [81] suggesting that two high ranked females could remain in close association feeding at desirable food sites. Interestingly, this is in contrast to previous studies in Bwindi mountain gorillas where high ranked females were less likely to have a partner feeding in close proximity [56]. Examining association scores in different contexts in the future could help to disentangle this contrast between populations and studies. For example, male–female affiliative relationships are considered key cornerstones of gorilla society and so gaining access to the alpha male and building a close bond with him could provide further benefits for high ranked females [82]. Alternatively, the causality could be in the opposite direction, high ranked females are more likely to gain access to high-quality resources and so by default are more likely to associate more frequently. Future investigation of male–female affiliative relationships can help to resolve this quandary.

Females dispersing among social groups multiple times in their lives is thought to be a mechanism of female choice to select for a high-quality mate [49,83,84]. However, it appears these dispersal events may come with social costs as we found that newly immigrated females were more likely to have weaker associations than other group members and likewise if one dyad member was new to a social group then the dyad had weaker association scores. Female gorillas may require a period to integrate into a new group [77]. Immigrant female gorillas are recipients of higher rates of intrasexual aggression [55] and so a new immigrant may keep her distance to avoid aggressive encounters. New immigrants may avoid approaching unknown individuals to within striking distance (5 m in our study) to reduce the risk of aggression, harassment and/or potential injury. Conversely, female gorillas may be neophobic, with unknown individuals being wary each other and a certain timeframe is required to build up trust/familiarity before association occurs. Furthermore, females that recently joined a group will not have a young infant, nor be high ranking, the two variables we found linked to higher/stronger female association scores.

Examining our demographic data (table 1), some behaviours were not expressed across all groups. In particular, the western gorilla group (ATA) had no new immigrant females join the group or alpha male turnovers but many female emigrations, unlike two mountain gorilla groups (ORU and BIT). This highlights the huge variation within and between groups/species and thus such longitudinal studies are vital to get to the crux of these socio-ecological phenomena. The variability in all demographic behaviours in the KYA group (observed for 18 years) emphasizes the importance of such long-term studies. Continued research into mountain and especially additional habituated western gorilla groups in the future is imperative and valuable to fully break down these species and group differences observed.

Taken together our results show that female mountain and western gorillas are using short-term contingencies to form short- to mid-term associations to fit to specific needs [36]. These needs could be related to risk aversion to enhance infant survival or to increase resource-holding potential. Two particularly rare life history traits could be driving this: first, females disperse from their natal groups and so are unlikely to reside with kin; second, they are one of a few mammalian species to show secondary dispersal. Unlike in species with female philopatry [6,9,46], or where females emigrate only once at maturity [20,22], potential association/social partners in gorillas could depart at any time. Instead, female gorillas focus on maintaining strong social relationships with the alpha male, who provides protection against infanticide and outsider males and may be more important for female fitness [49,83,84]. Continuous partner swapping or uncertainty is costly in blue monkeys (*Cercopithecus mitis*), where females who were inconsistent in their top partners across multiple years had a greater risk of mortality [85]. This costly social hedge-betting [24] may result from the social costs of building multiple new relationships where the main commodity is allo-grooming. However, short-term relationships may be less costly in our gorilla populations where grooming is scant, and associations are mainly spatial. Overall, the factors driving the social benefits on fitness may vary among species depending on the complex array of dispersal patterns, kinship and reproductive strategies of both males and females. Females must remain flexible in their formation of associations and the degree of investment in social behaviour with each other. Therefore, investing in long-term affiliative relationships with other females could be counter-productive and mean that after a large temporal and energetic investment they are left without a close associate if they emigrate. Female gorillas may have adopted a flexible strategy whereby they associate with other females based on a necessity for similar needs, in line with the similarity hypothesis. As kin-related benefits and long-term reciprocity cannot govern associations, homophily and the knowledge that a partner has the same needs could provide a base for associations. A potential partner expressing similar traits would be unlikely to defect because they require the same resource/support, therefore further enhancing the opportunity to associate.

Our results highlight strategies related to short-term associations between female gorilla partners (2 years on average: [41]) based on similar traits. Such short-term contingencies could be well suited to the flexible life history of female gorillas, where they can emigrate multiple times in their lifetimes and thus forming long-term strategic alliances seen in philopatric or one-time dispersers would prove a risky strategy. Therefore, flexibility in partner choice based on real-time necessities and contingencies as well as a degree of neophobia may be driving female gorilla associations. Modern human friendships are considered to be governed by homophily, and individuals with similar traits are much more likely to form friendships than dissimilar individuals [40,41]. The flexibility of modern society could echo that of gorilla group membership, with group members having the potential to leave without notice. Early hominid females are considered to have shown a similar group structure to gorillas [31,32,86,87] and so it may be that the tendency to associate with individuals with similar needs or traits is entrenched in our evolutionary history, which suggests that our understanding of female gorilla social structure can enhance our knowledge of the evolutionary origins of both ancestral and modern human social relationships.

## Data Availability

All code is archived at Zenodo [88] and data are available at Dryad [89]. Supplementary material is available online [90].
